# The Role of Protein Adduction in Toxic Neuropathies of Exogenous and Endogenous Origin

**DOI:** 10.3390/toxics9050098

**Published:** 2021-04-29

**Authors:** Peter S. Spencer, Xiao Chen

**Affiliations:** 1 Department of Neurology, School of Medicine, and Oregon Institute of Occupational Health Sciences, Oregon Health & Science University, Portland, OR 97239, USA; 2 Key Laboratory of Modern Toxicology of Shenzhen, Shenzhen Medical Key Subject of Health Toxicology (2020–2024), Shenzhen Center for Disease Control and Prevention, Shenzhen 518055, China; chenxiao@szcdc.net

**Keywords:** γ-diketone, amino acid pyrrolation, advanced glycation end product, protein cross-linking, central-peripheral distal axonopathy, diabetic neuropathy, uremic neuropathy, aging

## Abstract

The peripheral (axonal) neuropathy associated with repeated exposure to aliphatic and aromatic solvents that form protein-reactive γ-diketones shares some clinical and neuropathological features with certain metabolic neuropathies, including type-II diabetic neuropathy and uremic neuropathy, and with the largely sub-clinical nerve damage associated with old age. These conditions may be linked by metabolites that adduct and cross-link neuroproteins required for the maintenance of axonal transport and nerve fiber integrity in the peripheral and central nervous system.

## 1. Introduction

This paper discusses the presence of neurotoxic γ-diketones in normal subjects and in peripheral neuropathies resulting from overexposure to certain hydrocarbon solvents. Also considered is the possibility that γ-diketones of endogenous origin contribute to the etiology and pathogenesis of certain metabolic neuropathies, including type-II diabetic neuropathy and uremic neuropathy, and to the largely subclinical peripheral nerve changes that accumulate with age.

## 2. γ-Diketone Neuropathy

### 2.1. Clinical Picture

Repeated occupational exposure to organic solvent mixtures containing *n*-hexane or 2-hexanone, the common metabolite of which is the γ-diketone 2,5-hexanedione, is an established cause of sensory or sensory-motor neuropathy that has been reported in the printing, textile, furniture, auto, pharmaceutical, and shoemaking industries [1,2,3,4,5,6,7,8,9,10,11,12,13,14,15,16]. Mainly motor neuropathy also results from deliberate inhalation of *n*-hexane fumes [17,18,19,20,21,22]. Sensory abnormalities (pain, touch, vibration, temperature discrimination) and diminished deep tendon reflexes and progressive muscle weakness affect the distal extremities. Electrophysiological studies demonstrate prolongation of distal latencies, slowing of nerve conduction velocities, and conduction block with temporal dispersion in subjects with severe neuropathy. Sural nerve biopsy reveals 10 nm neurofilament-filled focal giant axonal swellings, secondary demyelination, and loss of large-diameter myelinated fibers. The evolution of symptoms and signs continues for a few weeks/months after exposure ceases, a phenomenon known as coasting, and this is followed by a slow recovery over subsequent months/years [17]. Severely affected patients may develop sequelae of muscle wasting, foot drop, and spasticity [12].

### 2.2. Experimental Animal Neuropathy

The human disease is readily and accurately reproduced in animals (rats, cats) treated systemically with aliphatic C6 compounds that form γ-diketone metabolites [23,24,25]. In rats treated subcutaneously with 2,5-hexanedione, the onset of clinical signs is insidious and occurs symmetrically in the hindlimbs. Nerve fiber degeneration is prominent in the distal tibial nerve and its branches, with few changes in more proximal regions. Lumbosacral motor neurons and dorsal root ganglion cells appear histologically normal. Myelinated tibial nerve fibers show focal distal axonal swellings, often on the proximal sides of nodes of Ranvier, secondary demyelination and remyelination, and distal atrophy. Axonal swellings of myelinated and unmyelinated axons contain excessive numbers of 10 nm neurofilaments. Distal regions with advanced nerve fiber degeneration are associated with endoneurial and sub-perineurial edema [26,27,28]. In the spinal cord/medulla oblongata, swollen axons are most prominent in the gracile nucleus, although in animals with early neuropathy, similar pathological changes are evident in the cuneate nucleus and in a superficial ventrolateral zone corresponding to dorsal and ventral spinocerebellar tracts. These findings demonstrate that γ-diketone neuropathy is a central-peripheral distal axonopathy [24,29].

**Figure 1:**
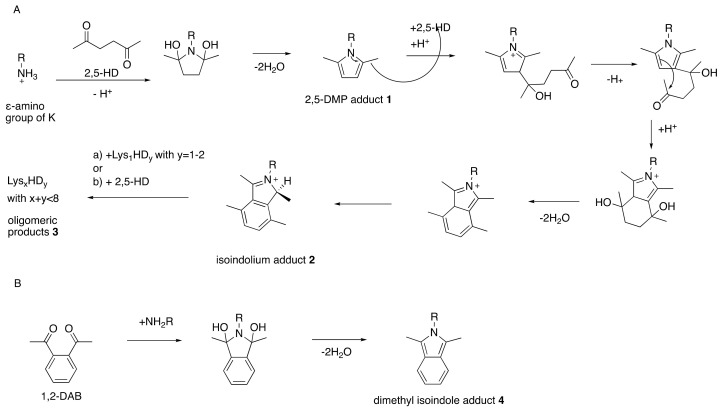
Interconvertibility of aliphatic and aromatic γ-diketones and their respective reactions with amino groups to form pyrrole (**A**) and isoindolium (**B**) adducts, respectively. Structures 1, 2, and 4 are experimentally observed and 5–8 are hypothesized [37]. Rats treated with 1,2-diacetylbenzene (1,2-DAB) develop a bluish coloration whereas 2,5-hexanedione (2,5-HD)-treated animals do not. This can be explained by the extended aromatic system in the case of 8 compared to 5 [37]. Even though 2,5-HD-derived 2 has high structural similarities with 1,2-DAB-derived 4 and 7, the reaction of 2,5-HD-induced 3 will not hypothetically proceed to 8 in vivo. From Trimpin, S.; Hsu, V.L.; Spencer, P.S.; Deinzer, M.L. Time-dependent 2,5-hexanedione adduction of lysine residues to isoindolium cation. Unpublished [38].

A similar peripheral neuropathy is seen in rats treated repeatedly with 1,2-diethylbenzene or 1,2,4-triethylbenzene but not in animals comparably exposed to 1,3-diethylbenzene or 1,3,5-triethylbenzene, both of which cannot form a γ-diketone metabolite [30,31,32]. While aromatic as well as aliphatic compounds that form γ-diketones elicit experimental neuropathy (Figure 1), the neurotoxic potency and corresponding location of nerve fiber pathology vary substantially [33]. For example, 1,2-diacetylbenzene and 3,4-dimethyl-2,5-hexanedione have a higher neurotoxic potency than 2,5-hexanedione, and neuropathological changes in the former occur in proximal (intraspinal and spinal roots) instead of distal regions of myelinated axons [33,34,35,36].

### 2.3. Neurotoxic Properties of γ-Diketones

**Figure 2:**
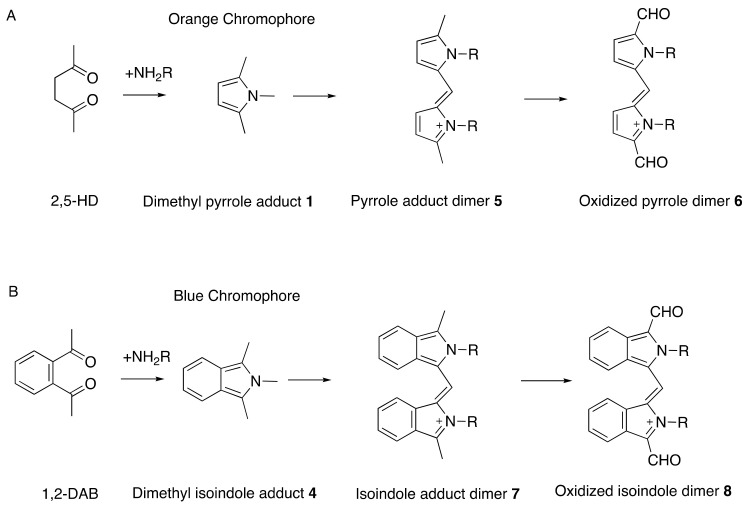
Reaction of 2,5-hexanedione (2,5-HD, (**A**)) and 1,2-diacetylbenzene (1,2-DAB, (**B**)) with amino residues in proteins to form dimethylpyrrole 1 and dimethyl isoindole products 4, respectively, that form colored oxidized polymers. More potent than 2,5-hexanedione is 3,4-dimethyl-2,5-hexanedione, with increased potential for pyrrole formation and protein crosslinking [35]; by contrast, 3,3-dimethyl-2,5-hexanedione, which cannot form a pyrrole, is unable to induce axonal neuropathy. Redrawn from Spencer, P.S. Neuroprotein targets of gamma-diketone metabolites of aliphatic and aromatic solvents that induce central-peripheral axonopathy. *Toxicol. Pathol.* **2020**, 48, 411–421, doi:10.1177/0192623320910960 [33]. By permission of Sage Publishing, 2021.

There is solid evidence that repeated exposure to aliphatic and aromatic compounds that generate γ-diketone metabolites induces distal symmetrical axonal neuropathy in humans and animals [33,39]. Aliphatic compounds are exemplified by straight-chain C6 solvents (such as *n*-hexane and 2-hexanone) that are metabolized to 2,5-hexanedione. This amine-reactive pyrrole-generating chromogenic (orange) γ-diketone is responsible for the induction of peripheral neuropathy in those repeatedly exposed to toxic levels of *n*-hexane-or 2-hexanone-containing solvents [40,41,42,43]. An important target of 2,5-hexanedione is the epsilon amino group of lysine residues in proteins, including neuroproteins. The pyrrole-forming, protein-crosslinking, and associated neurotoxic properties of 2,5-hexanedione are markedly enhanced by the addition of methyl groups at C3 and C4 [35,44,45], probably because molecular rotation is reduced, thereby promoting the critical γ-diketo-amine reaction. Even more theoretically rigid aromatic compounds, such as the potent neurotoxic solvent 1,2-diacetylbenzene, also react with proteins, generate a tissue chromogen (bluish), and precipitate proximal axonal neuropathy in laboratory animals [34,36,37,46]. Rodents treated systemically with 1,2-diacetylbenzene develop blue tissue discoloration prior to the development of hindlimb weakness. By contrast, animals treated with an aromatic isomer (1,3-diacetylbenzene) or aliphatic isomer (2,4-hexanedione) unable to react with amino groups of proteins to form pyrroles, fail to develop tissue discoloration or axonal neuropathy [36,47]. Thus, the protein reactivity, chromogenicity, and neurotoxicity of γ-diketones are directly related properties (Figure 2).

### 2.4. γ-Diketone Protein Adduction and Cross-Linking

Whereas 1,3-diacetylbenzene was non-chromogenic and non-neurotoxic, the widespread bluish discoloration of tissues of rats treated systemically with 1,2-diacetylbenzene indicated that the γ-diketone reacted with amino groups of proteins globally [46]. Measurement of chromogenic product in vitro showed that, of 22 tested amino acids, 1,2-diacetylbenzene was most reactive with L-lysine, glycine, γ-aminobutyric acid, and ornithine, a non-protein amino acid and higher homolog of L-lysine. L-Cysteine was among the least reactive with 1,2-diacetylbenzene, and 1,3-diacetylbenzene failed to react with any of the amino acids or with ornithine [46].

The protein reactivity of 1,2-diacetylbenzene and formation of adducts with high molecular weights correlated with the lysine content of neuronal proteins. Both in vivo and in vitro, rat neurofilament (NF) heavy chain (NF-H, 13–17% lysine) and NF-M (11–15% lysine) formed polymers more readily than either NF-L (6-7% lysine) or β-tubulin (3–4% lysine). Other 1,2-DAB protein targets involved in anterograde axonal transport included the rat motor proteins kinesin (8–12%), dynein (8% lysine) and microtubule-associated tau (7–8% lysine), and the rabbit glycolytic enzymes glyceraldehyde phosphate dehydrogenase (8% lysine) and lactate dehydrogenase (7–8% lysine) [46,48]. In vivo, 1,2-diacetylbenzene affected motor and cytoskeletal proteins in the sciatic nerve to a greater degree than in the spinal cord. The motor proteins responsible for anterograde axonal transport (kinesin) and retrograde transport (dynein), cytoskeletal protein NF-M, which is slowly transported from proximal to distal regions, and the microtubule-associated protein tau, were differentially impacted by 1,2-diacetylbenzene.

Neuroprotein changes in the lumbosacral spinal cord of rodents with 2,5-hexanedione axonopathy were comparable to those in animals with aromatic γ-diketone axonopathy induced by 1,2-diacetylbenzene [49,50]. Thirty-four proteins were markedly modified by both 2,5-hexanedione and 1,2-acetylbenzene, including neurofilament triplet L, gelsolin, protein disulfide isomerase, glutathione *S*-transferase, nicotinamide adenine dinucleotide (reduced) dehydrogenase 1α, pyruvate kinase, and fatty acid synthase. Proteins involved in energy metabolism were mostly increased, whereas those involved in cytoskeletal integrity or in redox and protein-folding mechanisms were reduced.

Proteomic studies showed that 1,2-diacetylbenzene, but not 1,3-diacetylbenzene, also reacted with mouse brain stathmin (15.5% lysine), which binds to tubulin and inhibits microtubule assembly [33,51]. Stathmin and proteins involved in stathmin regulation showed increased abundance in C57 mouse brain one week following single intraperitoneal treatment with 50 mg/kg 1,2-diacetylbenzene. At this time, marked 1,2-diacetylbenzene-specific changes were noted in the relative abundance of brain proteins involved in microtubule interaction (notably lysine-rich actin α−2) and energy metabolism, especially malate dehydrogenase [33], which also participates in gluconeogenesis. Noteworthy is that aged stathmin knock-out mice developed axonal degeneration and secondary demyelination [52] comparable with the neuropathology induced by blue-chromogenic acetyl-ethyltetramethyl tetralin [53]. Additionally, lysine-acetylated actin interacts with cyclase-associated protein to form an inhibitor of inverted formin 2, dominant missense mutations of which are linked with Charcot–Marie–Tooth disease, an inherited human axonal neuropathy associated specifically with glycolysis- and gluconeogenesis-related processes [54,55].

**Figure 3:**
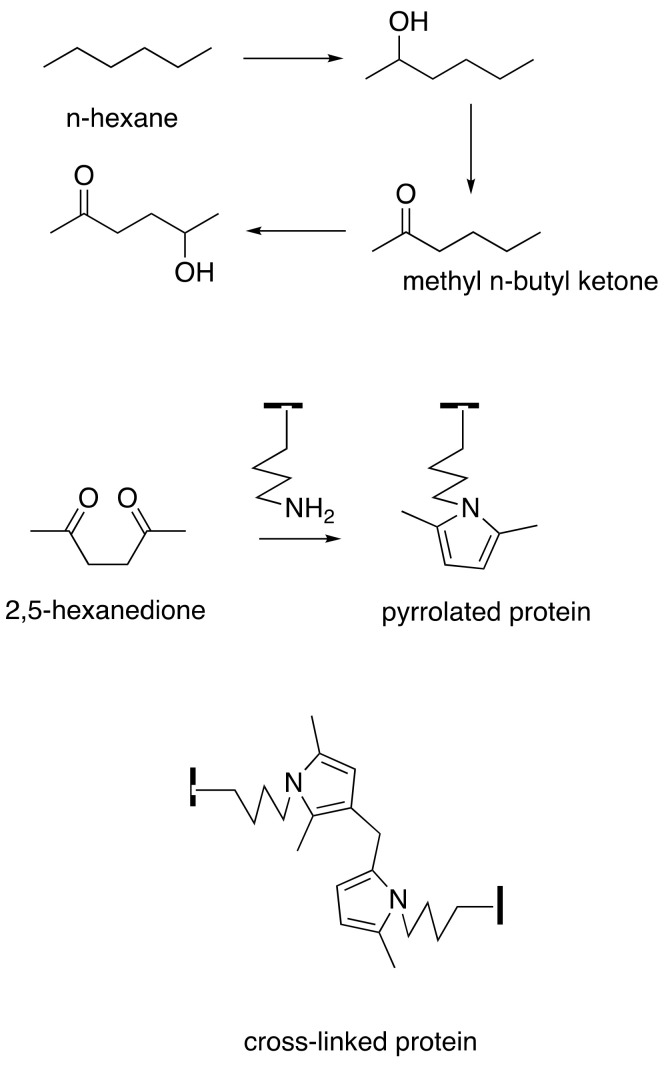
The C6 solvents *n*-hexane and methyl *n*-butyl ketone (2-hexanone) are converted by ω-1 hydroxylation and oxidation to 2,5-hexanedione, which reacts with lysyl ε-amines of proteins to form pyrrolated proteins that undergo intra- and intermolecular cross-linking reactions, including dimer formation. Redrawn from Boekelheide, K.; Fleming, S.L.; Allio, T.; Embree-Ku, M.E.; Hall, S.J.; Johnson, K.J.; Kwon, E.J.; Patel, S.R.; Rasoulpour, R.J.; Schoenfeld, H.A.; et al. 2,5-hexanedione-induced testicular injury. *Annu. Rev. Pharm. Toxicol.* **2003**, *43*, 125–147, doi:10.1146/annurev.pharmtox.43.100901.135930 [62]. By permission of Annual Reviews.

The vulnerability of proteins to γ-diketone attack is focused on their differential content of L-lysine, the epsilon-amino acid group of which is believed to be its primary vulnerability. 2,5-Hexanedione and 1,2-diacetylbenzene undergo selective Paal–Knorr reactions with lysine in axonal cytoskeleton proteins to form 2,5-dimethylpyrrole and 1,2-diacetylisoindole adducts, respectively [33,56] (Figure 2 and Figure 3). These adducts undergo progressive oxidative protein crosslinking, which can be inter- or intramolecular, depending on the adduct site [57,58]. Adduct oxidation is minimized in the absence of air or the presence of antioxidants [59,60], and hyperbaric oxygen accelerates the neurotoxicity of 2,5-hexanedione [61].

Rats treated orally with 2,5-hexanedione develop pyrrole adducts in hair, urine and serum in a dose–response relationship [63], and serum and urine pyrrole levels are correlated with time to onset of neuropathy induced by *n*-hexane administered to rats repeatedly by gavage [64]. The highest level of 2,5-hexanedione is found in urine and the lowest in the sciatic nerve, while the highest level of pyrrole adducts occurs in the liver and the lowest in serum. Pyrrole adducts in serum show the most significant correlation with free 2,5-hexanedione or pyrrole adducts in tissues [65].

### 2.5. Tissue Susceptibility to γ-Diketones

Systemic treatment with γ-diketones triggers pathological changes in the testes, as well as the nervous system. Extensive experimental studies of Sertoli cells in rats treated orally with 2,5-hexanedione point to alterations in microtubule-mediated functions, including microtubule assembly (which is associated with the status of stathmin phosphorylation), as well as kinesin and dynein motor-dependent transport [62,66]. The transport functions of axonal microtubules are also implicated in the genesis of nerve fiber degeneration. Injection of 2,5-hexanedione into the rat sciatic nerve results in rapid (< 30 sec) local reorganization of axoplasm, specifically the central accumulation of microtubules and peripheral relocation and disorientation of neurofilaments [67]. Microtubule cross-linking side arms are possible targets of lysine-reactive γ-diketones. Noteworthy is that spindle microtubule crosslinks contain PRC1 (protein regulator of cytokinesis 1), which binds microtubules via a structured domain with a spectrin-fold and an unstructured Lys/Arg-rich domain, and PRC1 shares the same binding site on the tubulin dimer as the motor proteins kinesin and dynein [68,69]. Clustered microtubules, sometimes associated with mitochondria, are often observed in the axons of peripheral nerve fibers of animals treated systemically with *n*-hexane, 2-hexanone, 2,5-hexanedione, or 1,2-diacetylbenzene [70,71], as well in a number of other experimental toxic neuropathies, human giant axonal neuropathy, and in hereditary distal motor and sensory neuropathy or Charcot-Marie-Tooth disease [72]. Since local 2,5-hexanedione nerve injection shows that the resulting local cytoskeletal reorganization precedes the formation of neurofilament-filled focal axonal swellings, microtubule displacement may mark sites at which anterograde transport of axonal neurofilaments is disrupted. Thus, intrathecal 2,5-hexanedione interfered with the slow axonal transport of neurofilaments [73], and systemic treatment with 3,4-dimethyl-2,5-hexanedione reduced the rate of transport of neurofilament proteins 75–90%, while tubulin and other proteins were only modestly retarded [74]. Similarly, anterograde neurofilament transport was slowed, but the velocities and amounts of labeled proteins in the fast anterograde component of the sciatic nerve were normal 2–5 h after eight once-weekly systemic treatments with 2,5-hexanedione [75]. Additionally, a single high intraperitoneal dose of 2,5-hexanedione produced a time-dependent decrease of fast retrograde transport velocity in mouse sciatic nerves (~65% inhibition between 2.0–2.5 h) with a reversal to normal rate 3–5 h after toxin administration, while retrograde transport velocity was halved in animals with hindlimb paralysis after repeated treatment with 3,4-dimethyl-2,5-hexanedione [76]. Motor nerves showed earlier and greater changes than sensory nerves in the accumulation of retrogradely transported materials [75,77].

Changes in anterograde neurofilament transport are consistent with initial nerve fiber changes in rats administered *n*-hexane, 2-hexanol, or 2,5-hexanedione. Teased fiber studies revealed that neurofilament-rich giant axonal swellings developed first on the proximal sides of multiple paranodes in distal non-terminal regions of large-diameter myelinated fibers. Paranodal myelin sheaths were often retracted at sites of axonal swellings, while adjacent distal internodes were attenuated. Axonal swellings later developed at internodal sites. Demyelinated paranodes apparently underwent local shrinkage and remyelination prior to the onset of distal nerve fiber breakdown. Smaller myelinated and unmyelinated fibers also contained multifocal, giant axonal swellings with abnormally large numbers of 10 nm neurofilaments. [70].

While neurofilaments are prominent in the pathological process of γ-diketone neuropathy, distal axonal degeneration also occurs in transgenic mice that lack axonal neurofilaments [78,79]. Taken in concert, these findings suggest that γ-diketones primarily disrupt microtubule-associated proteins (MAP) and MAP-microtubule binding [80], perhaps with preferential effect at nodes of Ranvier, and secondarily cause changes in bidirectional axonal transport associated with changes in neurofilament distribution that may result locally in axonal swelling or atrophy.

### 2.6. Summary

γ-Diketones react preferentially with lysine residues to form chromogenic oxidized pyrrole or isoindole adducts that can cross-link and disrupt the function of proteins, notably microtubule-associated proteins responsible for protein transport within cells. Neurons are vulnerable because they are required to transport proteins bidirectionally along elongate axons in the central and peripheral nervous system. γ-Diketone potency determines whether blockade of slow anterograde axonal transport of neurofilaments occurs proximally (3,4-dimethyl-2,5-hexanedione, 1,2-diacetylbenzene) or distally (2,5-hexanedione), with resulting focal neurofilament-filled axonal swellings that displace myelin sheaths resulting in localized demyelination and remyelination, nerve fiber and axonal attenuation and, eventually, distal axonal degeneration resulting in denervation of sensory end-organs and muscle fibers. Comparable changes occur later in smaller (and shorter) myelinated and unmyelinated nerve fibers. Contemporaneously, elongate ascending and descending axons in the spinal cord undergo comparable distal changes. Whereas, in humans, distal nerve fiber degeneration induced by aliphatic hexacarbons (*n*-hexane, 2-hexanone) manifests as a slowly ascending distal symmetrical peripheral neuropathy that may continue to evolve for some time after exposure ceases, sensation and muscle strength are restored in the opposite direction in association with axonal regeneration and end-organ reinnervation. However, failure of spinal motor pathways to regenerate may result in persistent spasticity in persons who have otherwise recovered from severe neuropathy [28,29]. The distal ends of other elongate nerve fibers in the central nervous system, including the optic nerve and mammillary bodies, may also develop pathological changes (neurofilament-filled axons) in γ-diketone intoxication [81,82,83,84].

## 3. Endogenous 2,5-Hexanedione and Associated Pyrroles

Human serum obtained from healthy American and European subjects contains detectable levels of compounds (2-hexanone, 3-heptanone) with the potential to form γ-diketones by ω-1 oxidation [85]. 2-Pentanone, 2- and 3-hexanone, 4-heptanone, and 5-methyl-2-hexanone were later identified in the human urinary metabolome [86]. Additionally, low levels of free 2,5-hexanedione are normally present in human serum (6–30 μg/L and urine (<12.0–77.9 μg/L), the latter representing 5th–95th percentiles of data collected from 99 Italian subjects [87,88]. The concentration of free 2,5-hexanedione tended to be higher in the urine of overweight subjects relative to those with normal body weight (*p* = 0.03) [88].

Larger amounts (*ca*. 0.45 mg/L) of 2,5-hexanedione were recovered from urine subjected to acid hydrolysis of samples from various urban and rural populations (Chinese, German, Japanese, Italian, Swedish) with no known exogenous exposure to *n*-hexane or 2-hexanone (Table 1). Levels of total and free (18% of total) urinary 2,5-hexanedione were correlated (r = 0.56; *p* < 0.0001) in such unexposed populations [88]. A similar percentage (mean = 14.2%) of free 2,5-hexanedione was found in the urine of 132 persons occupationally exposed to *n*-hexane [89]. γ–Diketone pyrroles have also been measured (1–6 μM) in healthy Chinese populations free of known exposure to these occupational hydrocarbon solvents.

These findings point to the endogenous production of reactive γ–diketones in healthy people, perhaps from lipid peroxidation [87,90] or other sources, including the endogenous production of *n*-hexane (and other straight-chain alkanes) from probable lipid peroxidation, as reported in humans and laboratory rats [104,105,106].

Urinary excretion of free 2,5-hexanedione did not seem to be influenced by gender (52M, 47F), age (39.6 ± 12.8 years, range: 17–67 years), smoking habit, or area of residence (urban vs. rural) [88]. Levels of acidified urinary 2,5-hexanedione were found to be similar in a small study of male and female Germans [90] but modestly higher in Chinese males than females [99]. Levels were somewhat higher in tobacco smokers but were apparently unaffected by diet or alcohol consumption [97,99]. Fluctuations typical of a circadian rhythm were not observed for 2,5-hexanedione in blood or urine. Excretion of 2,5-hexanedione was inversely related to age among Chinese subjects [99], while a comparison of two independent studies suggested that levels of urinary pyrroles were markedly higher in older persons [101,102].

## 4. Relationship to Neuropathies of Endogenous Origin

### 4.1. Central-Peripheral Distal Axonopathies

This term was introduced 45 years ago to describe the pathological substrate of distal symmetrical sensorimotor neuropathies (often with an autonomic component) that occur in a number of toxic, metabolic, nutritional, and inherited states [24,29]. The distal axonopathies comprise a group of diseases of varying etiology, of slow or rapid development, in which there is largely symmetrical axonal degeneration, commencing distally, spreading proximally, and rarely involving neuronal cell bodies. In the peripheral nervous system, secondary demyelination and remyelination are found proximal (sometimes including the spinal roots) to the region of active nerve fiber degeneration. On the central side, degenerative changes are symmetrical, tract-oriented, and involve, to a varying degree, the distal regions of the dorsal columns, especially the gracile tracts, the corticospinal tracts, and the spinocerebellar tracts. The central changes are usually clinically silent until post-exposure recovery from peripheral nerve damage is complete when, in severe cases, unrepaired and thus persistent damage to the central motor pathway may manifest in the form of lower-extremity hyperreflexia or even spasticity. Clinically silent features often include reduced or loss of vibratory perception that is greater in the lower than upper extremities, a result both of degenerative changes in nerve terminals innervating Pacinian corpuscles and in the terminal regions of their corresponding centrally projecting axons terminating in the gracile nucleus.

While the molecular etiology of peripheral neuropathies associated with central-peripheral distal axonopathy is often obscure, the exception being toxic γ-diketone neuropathy, certain peripheral neuropathies of metabolic origin are also associated with the endogenous production of molecules that form adducts with proteins and neuroproteins. Prominent among these metabolic disorders are type-II diabetes mellitus and, to a lesser extent, uremia, both of which are associated with distal symmetrical peripheral neuropathies that evolve as a consequence of metabolic imbalance. Similar molecular mechanisms may also have relevance to the etiology of age-related changes that resemble those found in mild central-peripheral distal axonopathy.

### 4.2. Type II-Diabetic Peripheral Neuropathy (T2DN)

#### 4.2.1. Clinical and Neuropathological Features

Distal symmetrical sensorimotor axonal neuropathy is the most common form of diabetic neuropathy [107,108], the incidence of which increases with age and duration of the diabetic state [109,110]. The prevalence of T2DN was 61.8% among Chinese patients (>30 years old, *n* = 435) seen in downtown Shanghai [111]. Most relevant here is the painless T2DN that manifests with impaired light touch sensation, position sense, vibratory perception, and diminished or absent ankle reflexes [112]. Sensorimotor polyneuropathy first affects the distal parts of the lower extremities and then spreads proximally [113]. Early loss of vibration perception is prominent in T2DN [114,115,116] and has a pivotal role in the early detection of nerve fiber degeneration [117]. The vibration perception threshold has high sensitivity and specificity relative to nerve conduction studies in the early detection of T2DN [118]. The neuropathology of T2BN conforms to a central-peripheral distal axonopathy [24,119].

Risk factors for diabetic neuropathy include age, male gender, height, duration of diabetes, uncontrolled glycemia, insulin resistance, and metabolic syndrome components, such as hypertriglyceridemia, hypertension, abdominal obesity, and low high-density lipoprotein level [109,120]. The pathogenesis of T2DN is not understood. Pathophysiological concepts include hypoxia, immune complexes, oxidative stress, reduced neurotrophic factors, mitochondrial dysfunction, inflammation, and inositol depletion leading to the accumulation of sorbitol and fructose [112,120,121].

#### 4.2.2. Glycation End-Products

**Figure 4:**
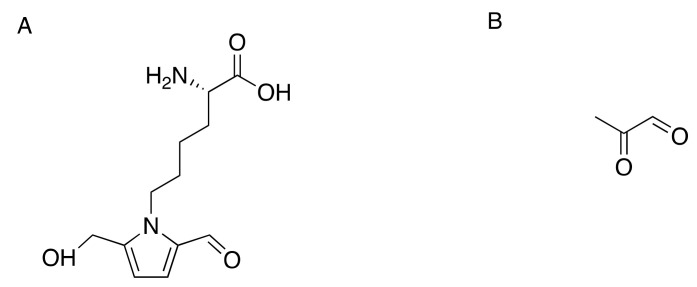
The structures of pyrraline, [2-formyl-5-(hydroxymethyl)pyrrole-1-norleucine] (**A**), and methylglyoxal (**B**).

Increased glucose levels in the diabetic state promote glycation of numerous structural and functional proteins. Glucose and other reducing sugars under physiological conditions can react nonenzymatically with free amino groups to form reversible Schiff base adducts (in days) and stable Amadori products (in weeks), which are then converted into advanced glycation end-products (AGE) through chemical rearrangements and degradation reactions [122]. Markedly elevated in diabetes mellitus are plasma levels of pyrraline (5-hydroxymethyl-1-alkylpyrrole-2-carbaldehyde) (Figure 4A), an AGE formed from 3-deoxyglucosone, the dicarbonyl product of a non-enzymatic reaction between glucose and the epsilon-amino group of lysine residues of proteins [123,124,125].

Additionally, the highly reactive dicarbonyl precursor methylglyoxal (Figure 4B), a byproduct of glycolysis resulting from the spontaneous degradation of the triosephosphates, dihydroxyacetone phosphate, and glyceraldehyde-3-phosphate [126,127,128], binds to arginine and promotes AGE formation. Methylglyoxal binds to lysine and cysteine residues in proteins to form *N*-α-acetyl derivatives [129]. The compound reacts irreversibly with lysine to form *N^ε^*-(1-carboxyethyl)lysine and the dimer 1,3-di(*N*^ε^-lysino)-4-methyl-imidazolium [130]. Methylglyoxal can also cross-link arginine and lysine residues resulting in the formation of 2-ammonio-6-((2-[(4-ammonio-5-oxido-5-oxopentyl)amino]-4-methyl-4,5-dihydro-1H-imidazol-5-ylidene)amino)hexanoate [131]. Elevated methylglyoxal, a marker of dicarbonyl stress, is a risk factor for type-2 diabetic neuropathy (T2DN) [132]. Analysis of reduced glycosylated amino acids in peripheral nerves showed that glycosylated lysine and its hydrolysis rearrangement products were more than doubled in diabetic rats and dogs compared with normal animals [133]. Additionally, tubulin and tubulin-associated proteins were readily modified by non-enzymatic glycosylation and cross-linking to form high-molecular aggregates [134].

#### 4.2.3. γ-Diketone-Derived Pyrroles

The genesis of diabetic neuropathy is associated with elevated levels of serum metabolites with the potential to form γ-diketones was first explored four decades ago [85]. Analysis of serum samples from subjects with and without diabetes mellitus revealed similar qualitative profiles of volatile metabolites (low nanogram concentration), with relatively high and low concentrations respectively of 2-hexanone and 3-heptanone, both of can undergo ω-oxidation to form γ-diketones, namely 2,5-hexanedione and 3,6-heptanedione [87,88]. Normal and diabetic sera contained similar concentrations of 2-butanone (methyl ethyl ketone), a compound that increases the persistence of blood-borne 2,5-hexanedione and markedly potentiates the neurotoxic potency of *n*-hexane and 2-hexanone [135,136,137]. Other aliphatic ketones found in normal and diabetic serum include 2-propanone, 2-pentanone, 4-heptanone, and 2-octanone [85]. Substances in the normal human urine metabolome include 2-pentanone, 2- and 3-hexanone, 5-methyl-2-hexanone [86,138], and 4-heptanone, which increases in diabetes mellitus and renal disease [139,140].

**Figure 5:**
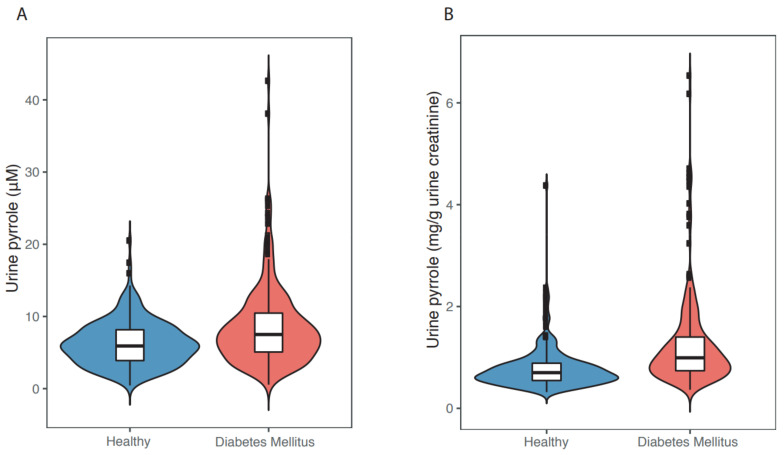
Violin plots of the distribution of urinary γ-diketone pyrrole adducts before (**A**) and after (**B**) correction for urinary creatinine, the excreted metabolite of muscle creatine. The white box in the center represents the interquartile range and the center line in the middle represents the median value. The thin black line extended from the thick black bar represents the upper (max) and lower (min) adjacent values of the data. (Figure 5A was redrawn from [102]).

In recent years, we and our colleagues have begun to examine more critically whether endogenous γ-diketones are involved in the genesis of T2DN. We assayed the urinary concentration of γ-diketone pyrroles in age- and gender-matched elderly (60–84 years) Chinese persons with and without (*n* = 267/group) indicators of diabetes mellitus. Subjects were drawn from a community of 9411 adults aged ≥ 60 years in the city of Shenzhen, Guangdong, China [102]. Subjects with diabetes mellitus relative to healthy controls had significantly higher levels of fasting blood glucose, glycated hemoglobin A1c, urinary ketone bodies, and urinary γ-diketone pyrroles. The median concentration of urinary γ-diketone pyrrole adducts (Figure 5) was significantly higher (*p* < 0.0001) in individuals with diabetes (7.5 μM or 0.99 mg/g urine creatinine) than in healthy controls (5.9 μM or 0.70 mg/g urine creatinine). Significant linear associations and non-linear associations were observed between urinary pyrrole adduct concentrations and diabetic indices (DM status, fasting blood glucose levels, or HbA1c levels). Studies are now underway to assess whether there is a correlation between urinary γ-diketone pyrroles and clinically measurable neuropathy.

In summary, from the time of onset of diabetes mellitus, glucose forms covalent adducts with plasma proteins through a non-enzymatic process known as glycation [141]. Protein glycation and formation of AGEs are considered to play an important role in the pathogenesis of diabetic complications, including neuropathy and retinopathy [142,143]. However, rats treated with intraperitoneal injection of AGE for 12 weeks failed to induce structural changes in tibial nerves other than interstitial edema [144]. Neuroprotein modifications triggered by endogenous levels of a pyrrole-forming γ-diketone might also participate in the genesis of T2DN.

### 4.3. Uremic Neuropathy

End-stage renal disease is commonly associated (16–60% of patients) with a slowly progressive symmetrical sensorimotor disorder of the axons of elongate large-diameter myelinated fibers [145]. Nerve conduction studies demonstrate findings consistent with a generalized neuropathy of the axonal type. Early clinical signs include distal loss of position and vibration sense plus a decreased Achilles reflex. Sensory deficits ascend to the knees, whereupon the hands may become involved. Advanced disease is associated with additional muscle atrophy and eventual paralysis. Patients may also develop autonomic features, with postural hypotension, impaired sweating, diarrhea, constipation, or impotence [145]. In sum, like T2DN, uremic neuropathy has features characteristic of a metabolic central-peripheral distal axonopathy.

The etiology of uremic neuropathy is unknown but has been related in part to elevated levels of blood urea [146]. Carbonyl stress may contribute to the long-term complications of chronic renal failure. AGEs (carboxymethyllysine and pentosidine) are markedly increased in plasma proteins and skin collagen of uremic patients, whether or not diabetes or elevated blood glucose is present [146,147,148]. Breath analysis of patients with end-stage renal disease undergoing dialysis included 4-heptanal, 4-heptanone, and 2-heptanone [149,150]. Another study of exhaled breath and blood identified 60 volatile organic compounds including *n*-alkanes (C4–C7), 2-ethyl-hexanol, 3-methyl-2-hexanone, 2-hexanone, and 3-heptanone, the latter two of which, plus the C6 alkane *n*-hexane, have the potential to form a neurotoxic γ-diketone.

### 4.4. Age-Associated Distal Axonopathy

By the seventh decade of life, most persons display evidence of peripheral nervous system dysfunction in the form of decreased vibration sensation, two-point discrimination, ankle reflexes, muscle bulk, and nerve conduction [151,152,153]. While the possible causes of these changes are manifold, including repeated trauma, focal nerve entrapment, and ischemia, the normal process of biological aging is thought to be an important contributing factor. The most suggestive evidence of central-peripheral distal axonopathy is the progressive increase in vibratory threshold with age, more in the lower limb (great toe) than in the upper limb (finger) [154,155,156,157,158,159,160]. Vibration perception also decreases with body height, which is consistent with a length-dependent axonopathy [160]. Other signs, such as distal impairment of tendon reflexes follow the same pattern in aging as in many distal axonopathies. Studies of very old rats reveal an age-associated axonopathy featured by axonal swelling and distal retrograde degeneration of the gracile tract, similar but less affected cuneate nuclei, spinocerebellar tracts, and corticospinal tracts [161]. While the etiology of these neuropathological changes is unknown, it is noteworthy that age-related changes are found in levels of 2,5-hexanedione (decrease) and associated pyrroles (increase) in acidified human urine [99,102]. This might indicate age-dependent increased binding of 2,5-hexanedione to target proteins, including neuroproteins responsible for maintenance of axonal integrity. AGEs are also formed in high amounts during aging [143,162,163,164,165].

## 5. Summary

The molecular mechanisms of peripheral neuropathy resulting from overexposure to certain organic solvents are largely understood. Several studies have shown that 2,5-hexanedione, the common γ-diketone metabolite of *n*-hexane and 2-hexanone, reacts with amino groups of proteins, including neuroproteins, to form pyrrolated products that undergo oxidation and cross-link proteins. Lysine-rich neuroproteins are particularly vulnerable, including microtubule-associated proteins required for axonal transport. Disruption of axonal transport results in sequential degenerative changes, notably focal neurofilament accumulation resulting in localized demyelination and remyelination, with initial changes in the largest and longest axons in peripheral nerves and the spinal cord, with similar changes in shorter nerve fibers at a later stage. This results in distal symmetrical sensorimotor neuropathy underpinned by central-peripheral distal axonopathy. Potent neurotoxic methylated aliphatic and aromatic γ-diketones disrupt axonal transport more proximally, which results in neurofilament blockade in spinal roots (leading to localized demyelination and remyelination) with distal nerve fiber atrophy.

Low levels of the γ-diketone 2,5-hexanedione appear to be a component of the normal metabolome. Unlike low tissue levels of other potentially neurotoxic substances with established physiological functions, such as carbon monoxide, cyanide, formaldehyde, and nitric oxide, endogenous 2,5-hexanedione has no known function. Urinary levels of 2,5-hexanedione seem to decline with the advance of age while its protein reaction product increases, which raises the possibility of an etiological relationship with the usually subclinical distal axonopathy associated with advancing age. Preliminary findings also show elevated urinary γ-diketone pyrroles in T2DM, a metabolic state that commonly results in a progressive distal symmetrical axonopathy. A similar length-dependent neuropathy develops in chronic renal disease, but tissue γ-diketone levels in this condition are unknown and uninvestigated. Other endogenous metabolites, notably methylglyoxal (a dicarbonyl with both keto and aldehyde groups), that bind to lysine and other protein residues have been demonstrated with the advance of age, in uremia, and especially in T2DM. However, pyralline was not detected (<1.2 pmol) in one study of serum albumin isolated from the serum samples of diabetic and non-diabetic subjects [166]. Further research is therefore needed to determine whether distal axonal degeneration in these conditions is triggered by the formation of advanced glycation products, 2,5-hexanedione-pyrrolated proteins, or both.

Note Added in Proof: Recently published data show that prolonged treatment of rats with large doses (400 mg/day, 5 days/week, for 5 weeks) of 2,5-hexanedione induces evidence of increased neuronal apoptosis in spinal anterior horn cells in association with severe limb weakness [167] indicative of marked central-peripheral distal axonopathy. Apoptotic changes in neuronal somata, which probably occur secondary to the onset of distal axonal degeneration/regeneration, involve changes in nerve growth factor and associated cell signal pathways [167,168,169].

## Figures and Tables

**Table 1:** Total * urinary 2,5-hexanedione (mg/L) and 2,5-dimethylpyrroles in populations with no known exposure to *n*-hexane or 2-hexanone.

*n*	Mean	Range	Method *	References
2,5-Hexanedione				
12 8M, 4F	0.45 ± 0.20	0.12–0.78	GC-FID	[90]
10M	0.49 ± 0.14	0.32–0.64	GC-FID	[91]
53M	0.33 ± 0.47	-	GC-FID	[92]
55M	1.47 ± 0.60	-		[93]
136 84M 52F	M+F: 0.35 M: 0.35 F: 0.49	-	GC-MS	[94]
26M	0.56 ± ?	0.17–0.98	GC-FID	[87]
40 20M, 20F	0.47 ± 0.21	0.20–1.00	GC-FID	[95]
22	0.44 ± 0.11	0.18–0.73	HPLC-F	[96]
123 60M + 63F	-	0.08–0.95	GC-FID HPLC-UV	[97]
110M 117F	M: 0.41 F: 0.38	-	GC	[98]
4216M 4019F	Median M: 0.171 F: 0.147		GC-MS	[99]
Pyrroles				
5M 5F 71.3 ± 5.4 years	-	-	ESI-LC-MS/MS	[100]
104M + 104F 18–24 years 21 ± 3 years	Median 0.91 μM		Chromatography	[101]
276M, 258F 68.3 ± 4.9 years	Median 7.4 μM		Chromatography	[102]

* After acid hydrolysis. ESI-LC-MS/MS Electrospray ionization liquid chromatography tandem mass spectrometry; GC-FID: Gas chromatography with mass-selective detection. GC-MS: Gas chromatography mass spectrometry using an ion-trap mass spectrometer. HPLC-F: High performance liquid chromatography (HPLC) with fluorescence detection. HPLC-UV: HPLC with UV detection. Acid hydrolysis converts other metabolites of *n*-hexane, such as 4,5-dihydroxy-2-hexanone, to 2,5-hexanedione [103].

## Data Availability

Data supporting Figure 5 are available on request from Xiao Chen.

## References

[1] Herskowitz A., Ishii N., Schaumburg H. (1971). N-hexane neuropathy. A syndrome occurring as a result of industrial exposure. N. Engl. J. Med..

[2] Ishii N., Herskowitz A., Schaumburg H.H. (1972). N-Hexane polyneuropathy: A clinical and experimental study. J. Neuropathol. Exp. Neurol..

[3] Mendell J.R., Saida K., Ganansia M.F., Jackson D.B., Weiss H., Gardier R.W., Chrisman C., Allen N., Couri D., O’Neill J. (1974). Toxic polyneuropathy produced by methyl N-butyl ketone. Science.

[4] Allen N., Mendell J.R., Billmaier D.J., Fontaine R.E., O’Neill J. (1975). Toxic polyneuropathy due to methyl n-butyl ketone. An industrial outbreak. Arch. Neurol..

[5] Mallov J.S. (1976). MBK neuropathy among spray painters. JAMA.

[6] Iida M. (1982). Neurophysiological studies of n-hexane polyneuropathy in the sandal factory. Electroencephalogr. Clin. Neurophysiol. Suppl..

[7] Wang J.D., Chang Y.C., Kao K.P., Huang C.C., Lin C.C., Yeh W.Y. (1986). An outbreak of N-hexane induced polyneuropathy among press proofing workers in Taipei. Am. J. Ind. Med..

[8] Chang C.M., Yu C.W., Fong K.Y., Leung S.Y., Tsin T.W., Yu Y.L., Cheung T.F., Chan S.Y. (1993). N-hexane neuropathy in offset printers. J. Neurol. Neurosurg. Psychiatry.

[9] Gluszcz-Zielinska A. (1999). Occupational N-hexane neuropathy: Clinical and neurophysiological investigation. Med. Pr..

[10] (2001). Centers for Disease Control and Prevention. n-Hexane-related peripheral neuropathy among automotive technicians—California, 1999–2000. MMWR. Morb. Mortal. Wkly. Rep..

[11] Puri V., Chaudhry N., Tatke M. (2007). N-hexane neuropathy in screen printers. Electromyogr. Clin. Neurophysiol..

[12] Huang C.C. (2008). Polyneuropathy induced by n-hexane intoxication in Taiwan. Acta Neurol. Taiwan.

[13] Misirli H., Domaç F.M., Somay G., Araal O., Ozer B., Adigüzel T. (2008). N-hexane induced polyneuropathy: A clinical and electrophysiological follow up. Electromyogr. Clin. Neurophysiol..

[14] Kim E.A., Kang S.K. (2010). Occupational neurological disorders in Korea. J. Korean Med. Sci..

[15] Pan J.H., Peng C.Y., Lo C.T., Dai C.Y., Wang C.L., Chuang H.Y. (2017). n-Hexane intoxication in a Chinese medicine pharmaceutical plant: A case report. J. Med. Case Rep..

[16] Neghab M., Soleimani E., Khamoushian K. (2012). Electrophysiological studies of shoemakers exposed to sub-TLV levels of n-hexane. J. Occup. Health.

[17] Goto I., Matsumura M., Inoue N., Murai Y., Shida K. (1974). Toxic polyneuropathy due to glue sniffing. J. Neurol. Neurosurg. Psychiatry.

[18] Shirabe T., Tsuda T., Terao A., Araki S. (1974). Toxic polyneuropathy due to glue-sniffing. Report of two cases with a light and electron-microscopic study of the peripheral nerves and muscles. J. Neurol. Sci..

[19] Korobkin R., Asbury A.K., Sumner A.J., Nielsen S.L. (1975). Glue-sniffing neuropathy. Arch. Neurol..

[20] Towfighi J., Gonatas N.K., Pleasure D., Cooper H.S., McCree L. (1976). Glue sniffer’s neuropathy. Neurology.

[21] Altenkirch H., Mager J., Stoltenburg G., Helmbrecht J. (1977). Toxic polyneuropathies after sniffing a glue thinner. J. Neurol..

[22] Tenenbein M., deGroot W., Rajani K.R. (1984). Peripheral neuropathy following intentional inhalation of naphtha fumes. Can. Med. Assoc. J..

[23] Schaumburg H.H., Spencer P.S. (1976). Degeneration in central and peripheral nervous systems produced by pure n-hexane: An experimental study. Brain.

[24] Spencer P.S., Schaumburg H.H., Zimmerman H.M. (1976). Central-peripheral distal axonopathy—The pathology of dying-back polyneuropathies. Progress in Neuropathology.

[25] Griffin J.W. (1981). Hexacarbon neurotoxicity. Neurobehav. Toxicol. Teratol..

[26] Spencer P.S., Schaumburg H.H. (1975). Experimental neuropathy produced by 2,5-hexanedione—A major metabolite of the neurotoxic industrial solvent methyl *n*-butyl ketone. J. Neurol. Neurosurg. Psychiatry.

[27] Politis M.J., Pellegrino R.G., Spencer P.S. (1980). Ultrastructural studies of the dying-back process. V. Axonal neurofilaments accumulate at sites of 2,5-hexanedione application: Evidence for nerve fibre dysfunction in experimental hexacarbon neuropathy. J. Neurocytol..

[28] Spencer P.S., Schaumburg H.H., Sabri M.I., Veronesi B. (1980). The enlarging view of hexacarbon neurotoxicity. Crit. Rev. Toxicol..

[29] Spencer P.S., Schaumburg H.H. (1977). Ultrastructural studies of the dying-back process. IV. Differential vulnerability of PNS and CNS fibers in experimental central-peripheral distal axonopathies. J. Neuropathol. Exp. Neurol..

[30] Gagnaire F., Ensminger A., Marignac B., De Ceaurriz J. (1991). Possible involvement of 1,2-diacetylbenzene in diethylbenzene-induced neuropathy in rats. J. Appl. Toxicol..

[31] Gagnaire F., Marignac B., de Ceaurriz J. (1993). Triethylbenzene-induced sensorimotor neuropathy in rats. J. Appl. Toxicol..

[32] Tshala-Katumbay D.D., Palmer V.S., Lasarev M.R., Kayton R.J., Sabri M.I., Spencer P.S. (2006). Monocyclic and dicyclic hydrocarbons: Structural requirements for proximal giant axonopathy. Acta Neuropathol..

[33] Spencer P.S. (2020). Neuroprotein targets of gamma-diketone metabolites of aliphatic and aromatic solvents that induce central-oeripheral axonopathy. Toxicol. Pathol..

[34] Anthony D.C., Boekelheide K., Graham D.G. (1983). The effect of 3,4-dimethyl substitution on the neurotoxicity of 2,5-hexanedione. I. Accelerated clinical neuropathy is accompanied by more proximal axonal swellings. Toxicol. Appl. Pharm..

[35] Anthony D.C., Boekelheide K., Anderson C.W., Graham D.G. (1983). The effect of 3,4-dimethyl substitution on the neurotoxicity of 2,5-hexanedione. II. Dimethyl substitution accelerates pyrrole formation and protein crosslinking. Toxicol. Appl. Pharm..

[36] Kim M.S., Sabri M.I., Miller V.H., Kayton R.J., Dixon D.A., Spencer P.S. (2001). 1,2-diacetylbenzene, the neurotoxic metabolite of a chromogenic aromatic solvent, induces proximal axonopathy. Toxicol. Appl. Pharm..

[37] Zhan C.-G., Spencer P., Dixon D.A. (2003). Computational insights into the chemical structures and mechanisms of the chromogenic and neurotoxic effects of aromatic γ-diketones. J. Phy. Chem. B.

[38] Trimpin S., Hsu V.L., Spencer P.S., Deinzer M.L. (2021). Time-dependent 2,5-hexanedione adduction of lysine residues to isoindolium cation.

[39] Spencer P.S., Kim M.S., Sabri M.I. (2002). Aromatic as well as aliphatic hydrocarbon solvent axonopathy. Int J. Hyg. Environ. Health.

[40] DeCaprio A.P. (1987). n-Hexane neurotoxicity: A mechanism involving pyrrole adduct formation in axonal cytoskeletal protein. Neurotoxicology.

[41] DeCaprio A.P., Briggs R.G., Jackowski S.J., Kim J.C. (1988). Comparative neurotoxicity and pyrrole-forming potential of 2,5-hexanedione and perdeuterio-2,5-hexanedione in the rat. Toxicol. Appl. Pharm..

[42] Genter M.B., Szakal-Quin G., Anderson C.W., Anthony D.C., Graham D.G. (1987). Evidence that pyrrole formation is a pathogenetic step in gamma-diketone neuropathy. Toxicol. Appl. Pharm..

[43] Ichihara G., Amarnath V., Valentine H.L., Takeshita T., Morimoto K., Sobue T., Kawai T., Valentine W.M. (2019). Pyrrole adducts in globin and plasma of workers exposed to hexane. Int. Arch. Occup. Environ. Health.

[44] Anthony D.C., Giangaspero F., Graham D.G. (1983). The spatio-temporal pattern of the axonopathy associated with the neurotoxicity of 3,4-dimethyl-2,5-hexanedione in the rat. J. Neuropathol. Exp. Neurol..

[45] Sanz P., Flores I.C., Soriano T., Repetto G., Repetto M. (1995). In Vitro quantitative structure-activity relationship assessment of pyrrole adducts production by gamma-diketone-forming neurotoxic solvents. Toxicol. In Vitro.

[46] Kim M.S., Hashemi S.B., Spencer P.S., Sabri M.I. (2002). Amino acid and protein targets of 1,2-diacetylbenzene, a potent aromatic gamma-diketone that induces proximal neurofilamentous axonopathy. Toxicol. Appl. Pharm..

[47] Spencer P.S., Bischoff M.C., Schaumburg H.H. (1978). On the specific molecular configuration of neurotoxic aliphatic hexacarbon compounds causing central—Peripheral distal axonopathy. Toxicol. Appl. Pharm..

[48] Sabri M.I., Hashemi S.B., Lasarev M.R., Spencer P.S. (2007). Axonopathy-inducing 1,2-diacetylbenzene forms adducts with motor and cytoskeletal proteins required for axonal transport. Neurochem. Res..

[49] Tshala-Katumbay D., Monterroso V., Kayton R., Lasarev M., Sabri M., Spencer P. (2008). Probing mechanisms of axonopathy. Part I: Protein targets of 1,2-diacetylbenzene, the neurotoxic metabolite of aromatic solvent 1,2-diethylbenzene. Toxicol. Sci..

[50] Tshala-Katumbay D., Monterroso V., Kayton R., Lasarev M., Sabri M., Spencer P. (2009). Probing mechanisms of axonopathy. Part II: Protein targets of 2,5-hexanedione, the neurotoxic metabolite of the aliphatic solvent n-hexane. Toxicol. Sci..

[51] Sabri M.I., Hashemi S.B., Chohan S., Cranson A.B., Tshala-Katumbay D.D., Palmer V.S., Pounds J.G., Spencer P.S. γ-Diketone toxicity: A role for stathmin in nerve and testes damage?. Proceedings of the Pacific Northwest Association of Toxicologists, 21st Annual Meeting.

[52] Liedtke W., Leman E.E., Fyffe R.E., Raine C.S., Schubart U.K. (2002). Stathmin-deficient mice develop an age-dependent axonopathy of the central and peripheral nervous systems. Am. J. Pathol..

[53] Spencer P.S., Sterman A.B., Horoupian D.S., Foulds M.M. (1979). Neurotoxic fragrance produces ceroid and myelin disease. Science.

[54] Bis-Brewer D.M., Danzi M.C., Wuchty S., Zuchner S. (2019). A network biology approach to unraveling inherited axonopathies. Sci. Rep..

[55] Mu A., Fung T.S., Kettenbach A.N., Chakrabarti R., Higgs H.N. (2019). A complex containing lysine-acetylated actin inhibits the formin INF2. Nat. Cell Biol..

[56] Kornienko A., La Clair J.J. (2017). Covalent modification of biological targets with natural products through Paal-Knorr pyrrole formation. Nat. Prod. Rep..

[57] DeCaprio A.P., Olajos E.J., Weber P. (1982). Covalent binding of a neurotoxic n-hexane metabolite: Conversion of primary amines to substituted pyrrole adducts by 2,5-hexanedione. Toxicol. Appl. Pharm..

[58] Graham D.G., Szakal-Quin G., Priest J.W., Anthony D.C. (1984). In Vitro evidence that covalent crosslinking of neurofilaments occurs in gamma-diketone neuropathy. Proc. Natl. Acad. Sci. USA.

[59] DeCaprio A.P. (1986). Mechanisms of in vitro pyrrole adduct autoxidation in 2,5-hexanedione-treated protein. Mol. Pharm..

[60] Kim M.S., Kim M.K., Kim K.S., Chung J.H., Kim S.J., Kim J.H., Kim J.R., Lee J., Yu B.P., Chung H.Y. (2008). Cytotoxicity of 1,2-diacetylbenzene in human neuroblastoma SHSY5Y cells is mediated by oxidative stress. Toxicology.

[61] Rosenberg C.K., Anthony D.C., Szakal-Quin G., Genter M.B., Graham D.G. (1987). Hyperbaric oxygen accelerates the neurotoxicity of 2,5-hexanedione. Toxicol. Appl. Pharm..

[62] Boekelheide K., Fleming S.L., Allio T., Embree-Ku M.E., Hall S.J., Johnson K.J., Kwon E.J., Patel S.R., Rasoulpour R.J., Schoenfeld H.A. (2003). 2,5-hexanedione-induced testicular injury. Annu. Rev. Pharm. Toxicol..

[63] Li X., Wang Q., Li M., Wang S., Zhang C., Xie K. (2018). Hair pyrrole adducts serve as biomarkers for peripheral nerve impairment induced by 2,5-hexanedione and n-hexane in rats. PLoS ONE.

[64] Yin H., Zhang C., Guo Y., Shao X., Zeng T., Zhao X., Xie K. (2014). Biological exposure indices of pyrrole adducts in serum and urine for hazard assessment of n-hexane exposure. PLoS ONE.

[65] Yin H., Guo Y., Zeng T., Zhao X., Xie K. (2013). Correlation between levels of 2, 5-hexanedione and pyrrole adducts in tissues of rats exposure to n-hexane for 5-days. PLoS ONE.

[66] Hall E.S., Hall S.J., Boekelheide K. (1995). 2,5-Hexanedione exposure alters microtubule motor distribution in adult rat testis. Fundam. Appl. Toxicol..

[67] Zagoren J.C., Politis M.J., Spencer P.S. (1983). Rapid reorganization of the axonal cytoskeleton induced by a gamma diketone. Brain Res..

[68] Subramanian R., Wilson-Kubalek E.M., Arthur C.P., Bick M.J., Campbell E.A., Darst S.A., Milligan R.A., Kapoor T.M. (2010). Insights into antiparallel microtubule crosslinking by PRC1, a conserved nonmotor microtubule binding protein. Cell.

[69] Kellogg E.H., Howes S., Ti S.C., Ramirez-Aportela E., Kapoor T.M., Chacon P., Nogales E. (2016). Near-atomic cryo-EM structure of PRC1 bound to the microtubule. Proc. Natl. Acad. Sci. USA.

[70] Spencer P.S., Schaumburg H.H. (1977). Ultrastructural studies of the dying-back process. III. The evolution of experimental peripheral giant axonal degeneration. J. Neuropathol. Exp. Neurol..

[71] Tshala-Katumbay D.D., Palmer V.S., Kayton R.J., Sabri M.I., Spencer P.S. (2005). A new murine model of giant proximal axonopathy. Acta Neuropathol..

[72] Xue C., Shtylla B., Brown A. (2015). A stochastic multiscale model that explains the segregation of axonal microtubules and neurofilaments in neurological diseases. PLoS Comput. Biol..

[73] Hammond-Tooke G.D. (1992). Slow axonal transport is impaired by intrathecal 2,5-hexanedione. Exp. Neurol..

[74] Griffin J.W., Anthony D.C., Fahnestock K.E., Hoffman P.N., Graham D.G. (1984). 3,4-Dimethyl-2,5-hexanedione impairs the axonal transport of neurofilament proteins. J. Neurosci..

[75] Braendgaard H., Sidenius P. (1986). Anterograde components of axonal transport in motor and sensory nerves in experimental 2,5-hexanedione neuropathy. J. Neurochem..

[76] Sabri M.I. (1992). Effect of 2,5-hexanedione and 3,4-dimethyl-2,5-hexanedione on retrograde axonal transport in sciatic nerve. Neurochem. Res..

[77] Braendgaard H., Sidenius P. (1986). The retrograde fast component of axonal transport in motor and sensory nerves of the rat during administration of 2,5-hexanedione. Brain Res..

[78] Stone J.D., Peterson A.P., Eyer J., Oblak T.G., Sickles D.W. (1999). Axonal neurofilaments are nonessential elements of toxicant-induced reductions in fast axonal transport: Video-enhanced differential interference microscopy in peripheral nervous system axons. Toxicol. Appl. Pharm..

[79] Stone J.D., Peterson A.P., Eyer J., Oblak T.G., Sickles D.W. (2001). Neurofilaments are nonessential to the pathogenesis of toxicant-induced axonal degeneration. J. Neurosci..

[80] Zhang L., Gavin T., DeCaprio A.P., LoPachin R.M. (2010). Gamma-diketone axonopathy: Analyses of cytoskeletal motors and highways in CNS myelinated axons. Toxicol. Sci..

[81] Schaumburg H.H., Spencer P.S. (1978). Environmental hydrocarbons produce degeneration in cat hypothalamus and optic tract. Science.

[82] Chang Y.C. (1987). Neurotoxic effects of n-hexane on the human central nervous system: Evoked potential abnormalities in n-hexane polyneuropathy. J. Neurol. Neurosurg. Psychiatry.

[83] Thomas P.K., Bradley D.J., Bradley W.A., Degen P.H., Krinke G., Muddle J., Schaumburg H.H., Skelton-Stroud P.N., Thomann P., Tzebelikos E. (1984). Correlated nerve conduction, somatosensory evoked potential and neuropathological studies in clioquinol and 2,5-hexanedione neurotoxicity in the baboon. J. Neurol. Sci..

[84] Chang Y.C. (1991). An electrophysiological follow up of patients with n-hexane polyneuropathy. Br. J. Ind. Med..

[85] Zlatkis A., Poole C.F., Brazeli R., Bafus D.A., Spencer P.S. (1980). Volatile metabolites in sera of normal and diabetic patients. J. Chromatogr..

[86] Bouatra S., Aziat F., Mandal R., Guo A.C., Wilson M.R., Knox C., Bjorndahl T.C., Krishnamurthy R., Saleem F., Liu P. (2013). The human urine metabolome. PLoS ONE.

[87] Perbellini L., Pezzoli G., Brugnone F., Canesi M. (1993). Biochemical and physiological aspects of 2,5-hexanedione: Endogenous or exogenous product?. Int. Arch. Occup. Environ. Health.

[88] Salamon F., Martinelli A., Trevisan A., Scapellato M.L., Bartolucci G.B., Carrieri M. (2019). Urinary Levels of free 2,5-hexanedione in Italian subjects non-occupationally exposed to n-hexane. App. Sci..

[89] Prieto M.J., Marhuenda D., Roel J., Cardona A. (2003). Free and total 2,5-hexanedione in biological monitoring of workers exposed to n-hexane in the shoe industry. Toxicol. Lett..

[90] Fedtke N., Bolt H.M. (1986). Detection of 2,5-hexanedione in the urine of persons not exposed to n-hexane. Int. Arch. Occup. Environ. Health.

[91] Perbellini L., Tagliaro F., Maschio S., Zedde A., Brugnone F. (1986). Gas chromatographic determination of 2,5-hexanedione in the urine. Med. Lav..

[92] Kawai T., Yasugi T., Mizunuma K., Horiguchi S., Uchida Y., Iwami O., Iguchi H., Ikeda M. (1991). Dose-dependent increase in 2,5-hexanedione in the urine of workers exposed to n-hexane. Int. Arch. Occup. Environ. Health.

[93] Kawai T., Mizunuma K., Yasugi T., Uchida Y., Ikeda M. (1990). The method of choice for the determination of 2,5-hexanedione as an indicator of occupational exposure to n-hexane. Int. Arch. Occup. Environ. Health.

[94] Sakai T., Araki T., Ushio K., Takeuchi Y., Ikeya Y. (1992). Effect of hydrolysis conditions on the determination of urinary 2,5-hexanedione in workers exposed or not exposed to N-hexane. Sangyo Igaku.

[95] Bavazzano P., Li Donni V., Baldasseroni A. (1993). Quality control in a system for the biological surveillance of exposure to n-hexane. Med. Lav..

[96] Maestri L., Ghittori S., Imbriani M., Capodaglio E. (1994). Determination of 2,5-hexandione by high-performance liquid chromatography after derivatization with dansylhydrazine. J. Chromatogr. B Biomed. Appl..

[97] Bavazzano P., Apostoli P., Balducci C., Bartolucci G.B., Buratti M., Duca P., Gori G., Li Donni V., Perbellini L., Perico A. (1998). Determination of urinary 2,5-hexanedione in the general Italian population. Int. Arch. Occup. Environ. Health.

[98] Persson B., Vrethem M., Murgia N., Lindh J., Hallsten A.L., Fredrikson M., Tondel M. (2013). Urinary 2,5-hexanedione excretion in cryptogenic polyneuropathy compared to the general Swedish population. J. Occup. Med. Toxicol..

[99] Pan X., Qian Y., Zhao W., Tang H., Ruan Z., Wu B., Huang H., Zheng Y., Yan H. (2016). Determination of total urinary 2,5-hexanedione in the Chinese general population. Environ. Res..

[100] Torres M.E. (2014). Characterization of Alternative Biomarkers to Control n-Hexane Exposure and Prevent 2,5-Hexanodione Toxicity.

[101] Wang H., Wang Y., Zhou Z., Wang S., Yin H., Xie K. (2015). Determination of normal reference value of pyrrole adducts in urine in young people in a university in Shandong, China. Zhonghua Lao Dong Wei Sheng Zhi Ye Bing Za Zhi.

[102] Chen X., Liu W., Wang L., Lin D., Nie L., He K., Guo Z., Zhu F., Feng W., Liu W. (2020). Diabetes mellitus is associated with elevated urinary pyrrole markers of gamma-diketones known to cause axonal neuropathy. BMJ Open Diabetes Res. Care.

[103] Dos Santos C.R., Meyer Passarelli M.M., de Souza Nascimento E. (2002). Evaluation of 2,5-hexanedione in urine of workers exposed to n-hexane in Brazilian shoe factories. J. Chromatogr. B Anal. Technol. Biomed. Life Sci..

[104] Sagai M., Ichinose T. (1980). Age-related changes in lipid peroxidation as measured by ethane, ethylene, butane and pentane in respired gases of rats. Life Sci..

[105] Kivits G.A., Ganguli-Swarttouw M.A., Christ E.J. (1981). The composition of alkanes in exhaled air of rats as a result of lipid peroxidation in vivo. Effects of dietary fatty acids, vitamin E and selenium. Biochim. Biophys. Acta.

[106] Gelmont D., Stein R.A., Mead J.F. (1981). Isoprene-the main hydrocarbon in human breath. Biochem. Biophys. Res. Commun..

[107] Sinnreich M., Taylor B.V., Dyck P.J. (2005). Diabetic neuropathies. Classification, clinical features, and pathophysiological basis. Neurologist.

[108] Feldman E.L., Callaghan B.C., Pop-Busui R., Zochodne D.W., Wright D.E., Bennett D.L., Bril V., Russell J.W., Viswanathan V. (2019). Diabetic neuropathy. Nat. Rev. Dis. Primers.

[109] Karvestedt L., Martensson E., Grill V., Elofsson S., von Wendt G., Hamsten A., Brismar K. (2011). The prevalence of peripheral neuropathy in a population-based study of patients with type 2 diabetes in Sweden. J. Diabetes Complicat..

[110] Simo N., Kuate-Tegueu C., Ngankou-Tchankeu S., Doumbe J., Maiga Y., Cesari M., Dartigues J.F., Kengne A.P., Tabue-Teguo M. (2020). Correlates of diabetic polyneuropathy of the elderly in Sub-Saharan Africa. PLoS ONE.

[111] Lu B., Yang Z., Wang M., Yang Z., Gong W., Yang Y., Wen J., Zhang Z., Zhao N., Zhu X. (2010). High prevalence of diabetic neuropathy in population-based patients diagnosed with type 2 diabetes in the Shanghai downtown. Diabetes Res. Clin. Pract..

[112] Hilz M.J., Marthol H., Neundorfer B. (2000). Diabetic somatic polyneuropathy. Pathogenesis, clinical manifestations and therapeutic concepts. Neurol. Psychiatr..

[113] Veves A., Sarnow M.R. (1995). Diagnosis, classification, and treatment of diabetic peripheral neuropathy. Clin. Podiatr. Med. Surg..

[114] Van der Naalt J., Fidler V., Oosterhuis H.J. (1991). Vibration perception threshold, complaints and sensory examination in diabetic patients. Acta Neurol. Scand..

[115] van Deursen R.W., Sanchez M.M., Derr J.A., Becker M.B., Ulbrecht J.S., Cavanagh P.R. (2001). Vibration perception threshold testing in patients with diabetic neuropathy: Ceiling effects and reliability. Diabet Med..

[116] Shen J., Zeng H., Li L., Bao Y., Liu F. (2013). The value of vibration perception threshold (VPT) in the diagnosis of diabetic peripheral neuropathy (DPN). Fudan Univ. J. Med. Sci..

[117] Medakkel A.A., Sheela P. (2018). Vibration perception threshold values and clinical symptoms of diabetic peripheral neuropathy. J. Clin. Diagn. Res..

[118] George A. (2013). Vibration Perception Threshold Measurements (VPT) in the Diagnosis of Diabetic Neuropathy. Ph.D. Thesis.

[119] Suzuki C., Ozaki I., Tanosaki M., Suda T., Baba M., Matsunaga M. (2000). Peripheral and central conduction abnormalities in diabetes mellitus. Neurology.

[120] Roman-Pintos L.M., Villegas-Rivera G., Rodriguez-Carrizalez A.D., Miranda-Diaz A.G., Cardona-Munoz E.G. (2016). Diabetic Polyneuropathy in type 2 diabetes mellitus: Inflammation, oxidative stress, and mitochondrial function. J. Diabetes Res..

[121] Greene D.A., Lattimer-Greene S., Sima A.A. (1989). Pathogenesis of diabetic neuropathy: Role of altered phosphoinositide metabolism. Crit. Rev. Neurobiol..

[122] Brownlee M., Cerami A., Vlassara H. (1988). Advanced glycosylation end products in tissue and the biochemical basis of diabetic complications. N. Engl. J. Med..

[123] Hayase F., Nagaraj R.H., Miyata S., Njoroge F.G., Monnier V.M. (1989). Aging of proteins: Immunological detection of a glucose-derived pyrrole formed during maillard reaction in vivo. J. Biol. Chem..

[124] Miyata S., Monnier V. (1992). Immunohistochemical detection of advanced glycosylation end products in diabetic tissues using monoclonal antibody to pyrraline. J. Clin. Investig..

[125] Portero-Otin M., Nagaraj R.H., Monnier V.M. (1995). Chromatographic evidence for pyrraline formation during protein glycation in vitro and in vivo. Biochim. Biophys. Acta.

[126] Bourajjaj M., Stehouwer C.D., van Hinsbergh V.W., Schalkwijk C.G. (2003). Role of methylglyoxal adducts in the development of vascular complications in diabetes mellitus. Biochem. Soc. Trans..

[127] Maessen D.E., Stehouwer C.D., Schalkwijk C.G. (2015). The role of methylglyoxal and the glyoxalase system in diabetes and other age-related diseases. Clin. Sci..

[128] Schalkwijk C.G., Stehouwer C.D.A. (2020). Methylglyoxal, a highly reactive dicarbonyl compound, in diabetes, its vascular complications, and other age-related diseases. Physiol. Rev..

[129] Lo T.W., Westwood M.E., McLellan A.C., Selwood T., Thornalley P.J. (1994). Binding and modification of proteins by methylglyoxal under physiological conditions. A kinetic and mechanistic study with N alpha-acetylarginine, N alpha-acetylcysteine, and N alpha-acetyllysine, and bovine serum albumin. J. Biol. Chem..

[130] Degenhardt T.P., Thorpe S.R., Baynes J.W. (1998). Chemical modification of proteins by methylglyoxal. Cell. Mol. Biol..

[131] Frye E.B., Degenhardt T.P., Thorpe S.R., Baynes J.W. (1998). Role of the Maillard reaction in aging of tissue proteins. Advanced glycation end product-dependent increase in imidazolium cross-links in human lens proteins. J. Biol. Chem..

[132] Andersen S.T., Witte D.R., Dalsgaard E.M., Andersen H., Nawroth P., Fleming T., Jensen T.M., Finnerup N.B., Jensen T.S., Lauritzen T. (2018). Risk factors for incident diabetic polyneuropathy in a cohort with screen-detected type 2 diabetes followed for 13 years: ADDITION-Denmark. Diabetes Care.

[133] Vlassara H., Brownlee M., Cerami A. (1981). Nonenzymatic glycosylation of peripheral nerve protein in diabetes mellitus. Proc. Natl. Acad. Sci. USA.

[134] Williams S.K., Howarth N.L., Devenny J.J., Bitensky M.W. (1982). Structural and functional consequences of increased tubulin glycosylation in diabetes mellitus. Proc. Natl. Acad. Sci. USA.

[135] Altenkirch H., Stoltenburg G., Wagner H.M. (1978). Experimental studies on hydrocarbon neuropathies induced by methyl-ethyl-ketone (MEK). J. Neurol..

[136] Veronesi B., Lington A.W., Spencer P.S. (1984). A tissue culture model of methyl ethyl ketone’s potentiation of n-hexane neurotoxicity. Neurotoxicology.

[137] Yu R.C., Hattis D., Landaw E.M., Froines J.R. (2002). Toxicokinetic interaction of 2,5-hexanedione and methyl ethyl ketone. Arch. Toxicol..

[138] Walker V., Mills G.A. (2001). Urine 4-heptanone: A beta-oxidation product of 2-ethylhexanoic acid from plasticisers. Clin. Chim. Acta.

[139] Liebich H.M., Woll J. (1977). Volatile substances in blood serum: Profile analysis and quantitative determination. J. Chromatogr..

[140] Liebich H.M. (1983). Gas chromatographic—Mass spectrometric determination of total 4-heptanone, a new marker in diabetes mellitus. J. Chromatogr..

[141] Lyons T.J., Jenkins A.J. (1997). Glycation, oxidation, and lipoxidation in the development of the complications of diabetes: A carbonyl stress hypothesis. Diabetes Rev..

[142] Singh V.P., Bali A., Singh N., Jaggi A.S. (2014). Advanced glycation end products and diabetic complications. Korean J. Physiol. Pharm..

[143] Wells-Knecht K.J., Brinkmann E., Wells-Knecht M.C., Litchfield J.E., Ahmed M.U., Reddy S., Zyzak D.V., Thorpe S.R., Baynes J.W. (1996). New biomarkers of Maillard reaction damage to proteins. Nephrol. Dial. Transplant..

[144] Nishizawa Y., Wada R., Baba M., Takeuchi M., Hanyu-Itabashi C., Yagihashi S. (2010). Neuropathy induced by exogenously administered advanced glycation end-products in rats. J. Diabetes Investig..

[145] Krishnan A.V., Kiernan M.C. (2007). Uremic neuropathy: Clinical features and new pathophysiological insights. Muscle Nerve.

[146] Anand U., Korchev Y., Anand P. (2019). The role of urea in neuronal degeneration and sensitization: An in vitro model of uremic neuropathy. Mol. Pain.

[147] Miyata T., van Ypersele de Strihou C., Kurokawa K., Baynes J.W. (1999). Alterations in nonenzymatic biochemistry in uremia: Origin and significance of “carbonyl stress” in long-term uremic complications. Kidney Int..

[148] Asahi K., Ichimori K., Nakazawa H., Izuhara Y., Inagi R., Watanabe T., Miyata T., Kurokawa K. (2000). Nitric oxide inhibits the formation of advanced glycation end products. Kidney Int..

[149] Pagonas N., Vautz W., Seifert L., Slodzinski R., Jankowski J., Zidek W., Westhoff T.H. (2012). Volatile organic compounds in uremia. PLoS ONE.

[150] Mochalski P., King J., Haas M., Unterkofler K., Amann A., Mayer G. (2014). Blood and breath profiles of volatile organic compounds in patients with end-stage renal disease. BMC Nephrol..

[151] Spencer P.S., Ochoa J., Johnson J.E. (1981). The mammalian peripheral nervous system in old age. Aging and Cell Structure.

[152] Schaumburg H.H., Spencer P.S., Ochoa J., Katzman R., Terry R. (1983). The aging peripheral nervous system. The Neurology of Aging.

[153] Lin Y.H., Hsieh S.C., Chao C.C., Chang Y.C., Hsieh S.T. (2005). Influence of aging on thermal and vibratory thresholds of quantitative sensory testing. J. Peripher. Nerv. Syst..

[154] Steiness I. (1957). Vibratory perception in normal subjects; a biothesiometric study. Acta Med. Scand..

[155] Rosenberg G. (1958). Effect of age on peripheral vibratory perception. J. Am. Geriatr. Soc..

[156] Perret E., Regli F. (1970). Age and the perceptual threshold for vibratory stimuli. Eur. Neurol..

[157] Verrillo R.T. (1980). Age related changes in the sensitivity to vibration. J. Gerontol..

[158] Meh D., Denislic M. (1995). Influence of age, temperature, sex, height and diazepam on vibration perception. J. Neurol. Sci..

[159] Deshpande N., Metter E.J., Ling S., Conwit R., Ferrucci L. (2008). Physiological correlates of age-related decline in vibrotactile sensitivity. Neurobiol. Aging.

[160] Nolte S., van Londen M., Elting J.W.J., de Greef B.T.A., Kuks J.B.M., Faber C.G., Nolte I.M., Groen R.J.M., Bakker S.J.L., Groothof D. (2020). Vibration threshold in non-diabetic subjects. PLoS ONE.

[161] Van Steenis G., Kroes R. (1971). Changes in the nervous system and musculature of old rats. Vet. Pathol..

[162] Nagaraj R.H., Portero-Otin M., Monnier V.M. (1996). Pyrraline ether crosslinks as a basis for protein crosslinking by the advanced Maillard reaction in aging and diabetes. Arch. Biochem. Biophys..

[163] Schleicher E.D., Wagner E., Nerlich A.G. (1997). Increased accumulation of the glycoxidation product N(epsilon)-(carboxymethyl)lysine in human tissues in diabetes and aging. J. Clin. Investig..

[164] Chellan P., Nagaraj R.H. (1999). Protein crosslinking by the Maillard reaction: Dicarbonyl-derived imidazolium crosslinks in aging and diabetes. Arch. Biochem. Biophys..

[165] Gkogkolou P., Bohm M. (2012). Advanced glycation end products: Key players in skin aging?. Dermato Endocrinol..

[166] Smith P.R., Somani H.H., Thornalley P.J., Benn J., Sonksen P.H. (1993). Evidence against the formation of 2-amino-6-(2-formyl-5-hydroxymethyl-pyrrol-1-yl)-hexanoic acid (‘pyrraline’) as an early-stage product or advanced glycation end product in non-enzymic protein glycation. Clin. Sci..

[167] Luo M., Shi X., Guo Q., Li S., Zhang Q., Sun X., Piao F. (2021). 2,5-Hexanedione induced apoptosis in rat spinal cord neurons and VSC4.1 cells via the proNGF/p75NTR and JNK pathways. Biosci. Rep..

[168] Wang Z., Qiu Z., Gao C., Sun Y., Dong W., Zhang Y., Chen R., Qi Y., Li S., Guo Y. (2017). 2,5-Hexanedione downregulates nerve growth factor and induces neuron apoptosis in the spinal cord of rats via inhibition of the PI3K/Akt signaling pathway. PLoS ONE.

[169] Zuo E., Zhang C., Mao J., Gao C., Hu S., Shi X., Piao F. (2019). 2,5-Hexanedione mediates neuronal apoptosis through suppression of NGF via PI3K/Akt signaling in the rat sciatic nerve. BioSci. Rep..

